# Development and validation of a nomogram to predict survival after curative resection of nonmetastatic colorectal cancer

**DOI:** 10.1002/cam4.3010

**Published:** 2020-04-21

**Authors:** Tingting Hong, Dongyan Cai, Linfang Jin, Ying Zhang, Tingxun Lu, Dong Hua, Xiaohong Wu

**Affiliations:** ^1^ Department of Medical Oncology The Affiliated Hospital of Jiangnan University and Wuxi 4th People's Hospital Wuxi China; ^2^ Department of Pathology The Affiliated Hospital of Jiangnan University and Wuxi 4th People's Hospital Wuxi China

**Keywords:** curative resection, nomogram, nonmetastatic colorectal cancer, overall survival

## Abstract

**Background:**

We aimed to develop a clinical applicable nomogram to predict overall survival (OS) for patients with curatively resected nonmetastatic colorectal cancer.

**Methods:**

Records from a retrospective cohort of 846 patients with complete information were used to construct the nomogram. The nomogram was validated in a prospective cohort of 379 patients. The performance of the nomogram was evaluated with concordance index (c‐index), time‐dependent receiver operating characteristic (ROC) curves, calibration plots, and decision curve analyses for discrimination, accuracy, calibration ability, and clinical net benefits respectively, and further compared with AJCC 8th TNM staging and the MSKCC nomogram. Risk stratification based on nomogram scores was performed with recursive partitioning analysis.

**Results:**

The nomogram incorporated age, Glasgow prognostic score, pretreatment carcinoembryonic antigen levels, T staging, N staging, number of harvested lymph nodes, and histological grade. Compared with the 8th AJCC staging and MSKCC model, the nomogram had a statistically higher c‐index (0.77, 95% CI: 0.73‐0.80), bigger areas under the time‐dependent ROC curves (AUC at 3 years: 79; at 5 years: 79), and improved clinical net benefits. Calibration plots revealed no deviations from reference lines. All results were reproducible in the validation cohort. Nomogram‐based risk stratification successfully discriminated patients within each AJCC stage (all log‐rank *P* < .05).

**Conclusion:**

We established an accurate, reliable, and easy‐to‐use nomogram to predict OS after curative resection for nonmetastatic colorectal cancer (CRC). The nomogram outperformed the 8th AJCC staging and the MSKCC model and could aid in personalized treatment and follow‐up strategy for CRC patients.

## Graphical abstract

We developed and validated a prognostic nomogram for non‐metastatic colorectal cancer incorporating routinely available factors. Nomogram based risk stratification successfully discriminated patients within each AJCC stage. The nomogram outperformed the 8th AJCC staging and the MSKCC model.

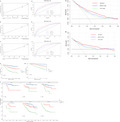

## 1 BACKGROUND

Colorectal cancer (CRC) is the third most commonly diagnosed and the second most deadly cancer in men and women worldwide.^1^ Surgical resection is the mainstay of treatment for patients with nonmetastatic CRC, and adjuvant treatment is recommended in high‐risk patients. The current gold standard for postoperation risk assessment is the TNM tumor staging system endorsed by the American Joint Commission on Cancer (AJCC). Although easy to implement with ordinal groups, survival outcomes within the same AJCC stage are quite heterogeneous due to the variability in clinicopathological features and tumor biology.^2^, ^3^ Survival paradox between stage IIB/C and stage IIIA patients is well recognized.^4^ Besides, in the era of precision medicine, the categorical TNM staging fails to provide individualized predictions. Risk calculators such as nomograms have gained popularity over classifiers such as TNM staging. The AJCC committee recognized the need to develop a prognostic tool to make more personalized probabilistic predictions than those conveyed by ordinal staging system and issued guidelines to develop nomograms incorporating additional anatomical and nonanatomical prognostic factors beyond TNM.^5^

Nomograms are statistical tools to provide the overall probability of a specific outcome by combining all proven prognostic variables. They utilize computational integration of multiple prognostic factors to quantify risks individually, rather than produce risk groups.^6^ Nomograms have been developed for a variety of malignancies for various outcome predictions. There are a few attempts for surgically resected CRC, but the overall quality is unsatisfactory.^7^ Most of the nomograms are developed from the population‐based database—the Surveillance Epidemiology and End Results (SEER) database.^8^, ^9^, ^10^ Although the sample size is quite large, a few well‐recognized prognostic factors are not incorporated. Clearly, more data elements are required than what is found in tumor registries.

Compared to tumor‐related factors, patient factors draw less attention. However, patient factors, for example, age, systemic inflammatory status, and nutritional status, are equally contributed to patients' prognosis.^11^ Data have shown that the most highly performing model includes tumor‐ and patient‐related factors.^10^ For years, systematic inflammatory status has been recognized as an important prognostic factor in cancer patients. A recent meta‐analysis revealed pretreatment Glasgow prognostic score (GPS) or modified Glasgow prognostic score (mGPS) is an independent prognostic predictor in CRC and could be useful in the management of CRC.^12^ To date, none of the nomograms incorporated GPS.

The goal of this project is to develop and assess a prognostic nomogram for curatively resected CRC by incorporating clinical available tumor‐ and patient‐related factors. We hope such a tool could help physicians to convey individualized survival information to every patient in daily practice without incurring additional cost.

## 2 METHODS

### 2.1 Patients

The data of patients with surgically treated nonmetastatic CRC patients were retrieved from a prospectively maintained cancer registry database of affiliated hospital of Jiangnan University as previously mentioned.^13^, ^14^ The last date of follow‐up was 28 December 2018.

Patients who received curative CRC surgery between 2008 and 2013 were as the primary cohort and those treated between 2014 and 2015 as the validation cohort. The inclusion criteria for the primary cohort were as follows: (a) Patients who had curative resection of primary CRC malignancies. (b) Who had histologically confirmed colorectal adenocarcinoma. (c) Who had the full blood cell count, biochemical profile, and tumor biomarker test at the hospital within 2 weeks before surgery. Patients with any of the following conditions were excluded: (a) Who had either imaging or histologically confirmed metastatic CRC diagnosed either preoperatively or intraoperatively. (b) Who had metastatic disease within 1 month after surgery. (c) Who had neo‐adjuvant chemotherapy, radiotherapy, or targeted therapy. (d) Who had bowel obstruction or perforation with emergency presentation. (e) Who was complicated with other acute diseases such as pneumonia, urinary tract infection, and cholecystitis. (f) Who had a history of chronic inflammatory diseases such as inflammatory bowel diseases and rheumatoid arthritis. (g) Who had a previous history of malignancies including CRC at different sites. (h) Who died within 1 month after surgery. (i). Whose survival status could not be ascertained. (j) Whose number of sampled lymph nodes was below 12.

The information for potential prognostic variables was collected: demographic characteristics including age and sex; pathological characteristics including primary site of tumor, histology, depth of primary tumor invasion (T), number of total lymph nodes sampled (TLN), number of metastasized lymph nodes (LNM), histological grade (G1‐4), the presence of peri‐neural invasion (PNI) and lymph‐vascular invasion (LVI) and number of tumor deposits (TDs); blood biomarkers including carcinoembryonic antigen (CEA), white blood cell count, neutrophil count, lymphocyte count, albumin, and C‐reactive protein (CRP). GPS was derived as previously stated.

Informed consent to the usage of social‐demographic and clinical information in scientific endeavors was obtained from every participant. This study was approved by the ethics review board of the hospital, adhered to the Declaration of Helsinki for medical research involving human subjects, and conducted according to the TRIPOD statement.^15^

### 2.2 Survival analysis and nomogram development

The endpoint for this study was overall survival (OS), which was defined as the time from the date of surgery to the date of death from any cause or the last date of follow‐up. Patients were censored if they were diagnosed with second malignancies after surgery. Only cases with complete information were used in the final analysis.

Normally distributed continuous variables were described as mean with standard error (SD), otherwise median values with interquartile ranges (IQR). Cox proportional hazards regression modeling was used to assess the relationship of OS with predictive variables. For continuous variables, possible nonlinearity effects on the log relative hazard of outcome were tested by modeling with restricted cubic splines, whereas statistically significant nonlinearity was identified, restricted cubic splines were used in the multivariable modeling. If restricted cubic spline modeling was failed, continuous variables were dichotomized, for which the optimal cut‐points were determined by the maximally selected rank statistics to maximize the correlation with survival. The proportional hazards assumption for each variable was checked by the test proposed by Grambsch and Therneau. Multivariate models were built by including all variables from univariate models (*P* < .2) in a backward stepwise selection with minimal AIC (the Akaike information criterion) value. Nomogram based on the final model was constructed for the likelihood of overall survival at 3 and 5 years of surgery.

### 2.3 Nomogram performance evaluation

Internal validation of the nomogram was achieved with bootstrap resampling strategy (1000 resamples). External validation was conducted in the prospective validation cohort. Briefly, the validation cohort was individually given a risk score calculated with the nomogram equation.

The performance of the nomogram was assessed and compared with the MSKCC model^10^ and the 8th AJCC TNM staging. The discrimination ability of the nomogram was evaluated with the concordance index (c‐index) and AIC value. A c‐index of 0.5 indicated a random chance and 1.0 indicated a perfect ability to correctly discriminate the outcome with the model. The smaller the AIC value, the more the goodness‐of‐fit of a model. The calibration ability of the nomogram was evaluated with calibration curves for 3‐ and 5‐year OS comparing the predicted survival with the observed survival. The predictive accuracy of the nomogram was quantified and compared using the area under the time‐dependent ROC curves (AUC). A recursive partitioning analysis (RPA) was conducted to categorize risk groups based on nomogram‐derived scores. Kaplan‐Meier survival curves and log‐rank tests were used to assess the risk stratification ability of the nomogram within AJCC stages. Finally, a decision curve analysis (DCA) was conducted to determine the clinical usefulness of the nomogram by quantifying the net benefits at different threshold probabilities.^16^

All statistical analyses were two sided with *P* < .05 as significant and conducted with Stata 14 or R studio software (version 1.1.456).

## 3 RESULTS

### 3.1 Patients characteristics

A total of 1576 patients were initially screened for enrollment eligibility in the study. After application of exclusion criteria, 836 patients with complete information were included in the final analysis for the primary cohort. For the validation cohort, a total of 379 patients of 505 patients were included in the final analysis after application of the same inclusion and exclusion criteria. The major reason for exclusion was less than 12 lymph nodes sampled, followed by metastasized disease and preoperative treatment. The percentage of patients excluded for missing data was less than 10%.

The baseline characteristics of the primary cohort and validation cohort were listed in Table 1. The characteristics were well balanced between the two cohorts.

**TABLE 1:** Demographic and clinical‐pathological characteristics of the primary cohort and validation cohort

	Primary cohort N = 846	Validation cohort N = 379
Follow‐up (mo)		
Median	52	43
Range	4‐132	5‐62
Number of events		
Dead/live	187/659	52/327
Age (y)		
Median	64	65
Interquartile	56‐72	57‐72
Range	19‐91	19‐89
Sex		
Male/female	489/407	225/154
GPS		
0/1/2	615/165/66	268/76/35
WBC (*10^9^/L)		
Median	6.1	6.0
Interquartile	5.0‐7.2	5.0‐7.3
Range	2.1‐26.4	2.1‐18
ABN (*10^9^/L)		
Median	3.7	3.6
Interquartile	2.9‐4.7	2.8‐4.8
Range	1.1‐23.6	1.1‐15.5
Lymphocyte (*10^9^/L)		
Median	1.6	1.6
Interquartile	1.3‐2.0	1.3‐2.0
Range	0.3‐7.9	0.5‐7.9
Albumin (g/L)		
Median	40.1	40.0
Interquartile	37.4‐43.0	36.9‐39.8
Range	24.8‐67.1	24.8‐52.2
CRP (mg/L)		
Median	4	4.0
Interquartile	2‐10.9	2.0‐10.5
Range	0‐143	1.0‐143
CEA (ng/mL)		
Median	3.3	3.3
Interquartile	1.9‐12.5	1.8‐7.0
Range	0.2‐1000	0.4‐285.1
Tumor location		
Left/right	621/225	265/106
T stage		
1/2/3/4a/4b	23/153/244/415/6	17/62/90/209/1
N stage		
0/1a/1b/1c/2a/2b	454/115/113/26/71/62	221/54/38/14/23/29
AJCC stage		
I/IIA/IIB/IIC/IIIA/IIIB/IIIC	130/138/186/2/36/251/103	62/58/101/0/13/103/42
Histological grade		
1/2/3/4	52/640/113/36	44/269/45/21
TLN		
Median	17	18
Interquartile	14‐21	15‐23
Range	12‐101	12‐101
LNM		
Range	0‐19	0‐17
Tumor deposits		
Range	0‐5	0‐6
Positive/negative	71/775	38/341
LVI		
Negative/positive/indeterminate	664/172/5	288/86/5
PNI		
Negative/positive/indeterminate	739/102/5	325/49/5

Note

Right‐sided colorectal cancers comprise cancers of the cecum, ascending and transverse colon up to the liver flexure, and left‐sided colorectal cancers comprise transverse colon distal to the liver flexure, descending and sigmoid colon and rectum.

Abbreviations: ABN, absolute neutrophil count; CEA, carcinoembryonic antigen; CRP, C‐reactive protein; GPS, Glasgow prognostic score; LNM, number of metastasized lymph nodes; LVI, lymph‐vascular invasion; PNI, peri‐neural invasion; TLN, number of total lymph nodes sampled; WBC, white blood cell count.

### 3.2 Survival analysis and development of the nomogram in the primary cohort

Among continuous variables, only age and LNM had linear effects (nonlinear *P* = .016 and <.001, respectively). Restricted cubic spline modeling was applied to all other continuous variables with nonlinear effects, except for CEA. CEA more than 20.03 ng/mL was regarded as high CEA level, otherwise as low. The results of univariate cox regression survival analysis for the primary cohort were presented in Table 2.

**TABLE 2:** Univariate and multivariate cox proportional hazards regression analysis in the primary cohort

	Univariate model^a^		Multivariate model^b^	
	HR (95% CI)	*P*	HR (95% CI)	*P*
Age	1.03 (1.01‐1.04)	<.001	1.02 (1.00‐1.03)	<.001
Sex				
Male	1.00			
Female	0.85 (0.63‐1.14)	.27		
GPS				
0	1.00		1.00	
1	1.99 (1.43‐2.77)	<.001	1.99 (1.41‐2.82)	<.001
2	2.56 (1.65‐3.95)	<.001	2.64 (1.68‐4.14)	<.001
WBC^c^		.4		
ABN^c^		.1		
Lymphocyte^c^		<.01		
Albumin^c^		<.001		
CRP^c^		<.001		
CEA (ng/mL)				
≤20.03	1.00		1.00	
>20.03	2.89 (2.06‐4.02)	<.001	2.68 (1.89‐3.80)	<.001
Location				
Left‐side	1.00			
Right‐side	1.13 (0.82‐1.56)	.4		
T				
1‐3	1.00		1.00	
4a	1.91 (1.42‐2.59)	<.001	1.34 (1.01‐1.84)	.03
4b	3.88 (1.22‐12.33)	<.001	3.94 (1.19‐1.31)	.01
N				
N0	1.00		1.00	
N1	2.24 (1.58‐3.16)	<.001	1.66 (1.17‐2.37)	<.01
N2a	2.52 (1.54‐4.10)	<.001	2.12 (1.29‐3.50)	<.01
N2b	5.91 (3.86‐9.05)	<.001	5.04 (3.11‐8.16)	<.001
Grade				
1	1.00		1.00	
2	2.46 (0.91‐6.64)	.07	1.96 (0.72‐5.37)	
3	3.52 (1.24‐9.99)	.02	2.23 (0.77‐6.41)	
4	8.27 (2.76‐24.76)	<.001	3.67 (1.19‐11.28)	<.01
TLN^c^		.02		<.001
LNM	1.14 (1.10‐1.18)	<.001		
TDs				
Negative	1.00			
Positive	1.88 (1.23‐2.87)	<.01		
LVI				
Negative	1.00			
Positive	1.80 (1.31‐2.47)	<.001		
PNI				
Negative	1.00			
Positive	2.49 (1.76‐3.52)	<.001		

Abbreviations: ABN, absolute neutrophil count; CEA, carcinoembryonic antigen; CRP, C‐reactive protein; GPS, Glasgow prognostic score; LNM, number of metastasized lymph nodes; LVI, lympho‐vascular invasion; PNI, peri‐neural invasion; TLN, number of total lymph nodes sampled; WBC, white blood cell count.

^a^ In univariate models, continuous variables with nonlinear effects such as WBC, ABN, lymphocyte, CRP, albumin, and TLN were modeled with restricted cubic splines. The significances were checked with log‐rank tests.

^b^ The final multivariate variable model selected with the minimal AIC value included TLN modeled with restricted cubic splines.

^c^ Restricted cubic spline modeling.

**FIGURE 1:**
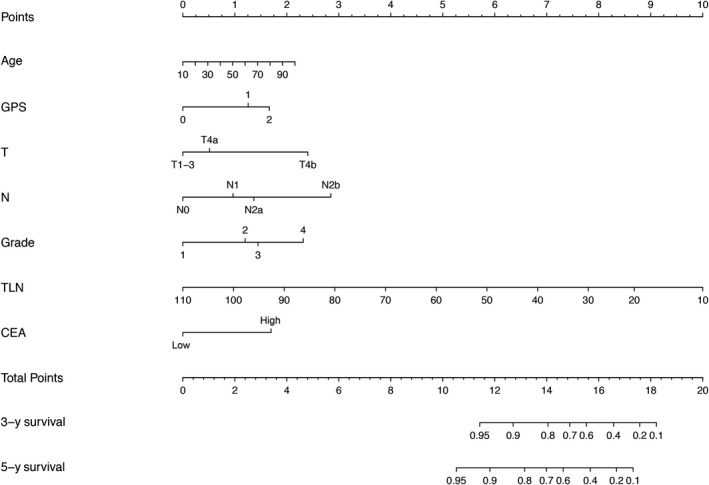
The nomogram to predict 3‐year and 5‐year survival probability for patients with curatively resected nonmetastatic colorectal cancer based on the primary cohort. Instructions for users: Locate the patient's age on the age axis. Draw a straight line up to the points axis to determine how many points the patient should receive. Repeat this process for each prognostic variable. Sum the points received for each variable and locate the number on the total points axis. Draw a straight line down from the total points to the 3‐ and 5‐year survival axis to determine the patient's individualized risk of remaining alive 3 and 5 y after surgery. Abbreviations: CEA, carcinoembryonic antigen; GPS, Glasgow prognostic score; TLN, number of total lymph nodes sampled

The final multivariate cox regression model with the minimal AIC value incorporated seven variables, including age, GPS, CEA, T staging, N staging, TLN, and histological grade (Table 2). A nomogram estimating 3‐ and 5‐year OS after curative surgery was developed incorporating the seven variables (Figure 1). A risk score was given for each case according to the nomogram equation.

### 3.3 Internal and external validation of the nomogram

For the internal validation, the bootstrap corrected c‐index for the nomogram was 0.77 (95% CI: 0.74‐0.80). For the external validation, the score for the individual case in the validation cohort was calculated according to the established nomogram and was then used in the Cox regression model. C‐index for the validation cohort was 0.79 (95% CI: 0.73‐0.85).

### 3.4 Calibration curves analysis of the nomogram

**FIGURE 2:**
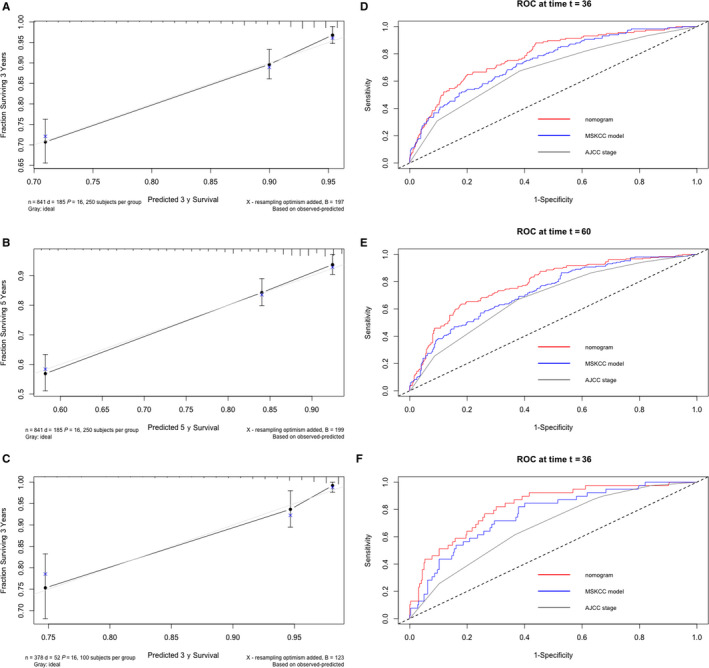
The calibration curves of 3‐ and 5‐year survival probability for the primary cohort (A, B) and the calibration curve of 3‐year survival probability for the validation cohort (C). Time‐dependent ROC analysis of the nomogram, MSKCC model and AJCC staging at 3‐ and 5‐year time point in the primary cohort (D, E) and 3‐year time point in the validation cohort (F)

The calibration plots showed a good agreement between observed and nomogram predicted 3‐ (Figure 2A) and 5‐year OS in the primary cohort (Figure 2B). The nomogram also demonstrated appreciable reliability in predicting 3‐year OS in the validation cohort (Figure 2C).

### 3.5 Comparison of the nomogram with the MSKCC model and AJCC staging

The nomogram had a statistically higher c‐index compared with the MSKCC model and AJCC staging in both the primary cohort and validation cohort. The c‐indexes were 0.77 (95% CI: 0.73‐0.80), 0.73 (95% CI: 0.70‐0.76), 0.69 (95% CI: 0.66‐0.92) for the nomogram, MSKCC model, and AJCC staging respectively in the primary cohort and 0.79 (95% CI: 0.73‐0.85), 0.75 (95% CI: 0.68‐0.81), 0.68 (95% CI: 0.61‐0.75) in the validation cohort. The nomogram had the lowest AIC value among the three models (2180, 2296, and 2321 for the nomogram, MSKCC model, and AJCC staging respectively in the primary cohort and 534, 566, 573 in the validation cohort).

In the primary cohort, for the nomogram model, MSKCC model and AJCC staging respectively, the AUC at the 3 years were 78.4, 72.9 and 68.3 (*P* < .05, all pair‐wise comparisons) (Figure 2D) and the AUC at the 5 years were 78.4, 72.7, 68.6 (*P* < .05, all pair‐wise comparisons) (Figure 2E). In the validation cohort, the AUC at 3 years were 82.3, 76.2, and 67.5 respectively for the nomogram model, MSKCC model, and AJCC staging (*P* < .05, all pair‐wise comparisons) (Figure 2F).

**FIGURE 3:**
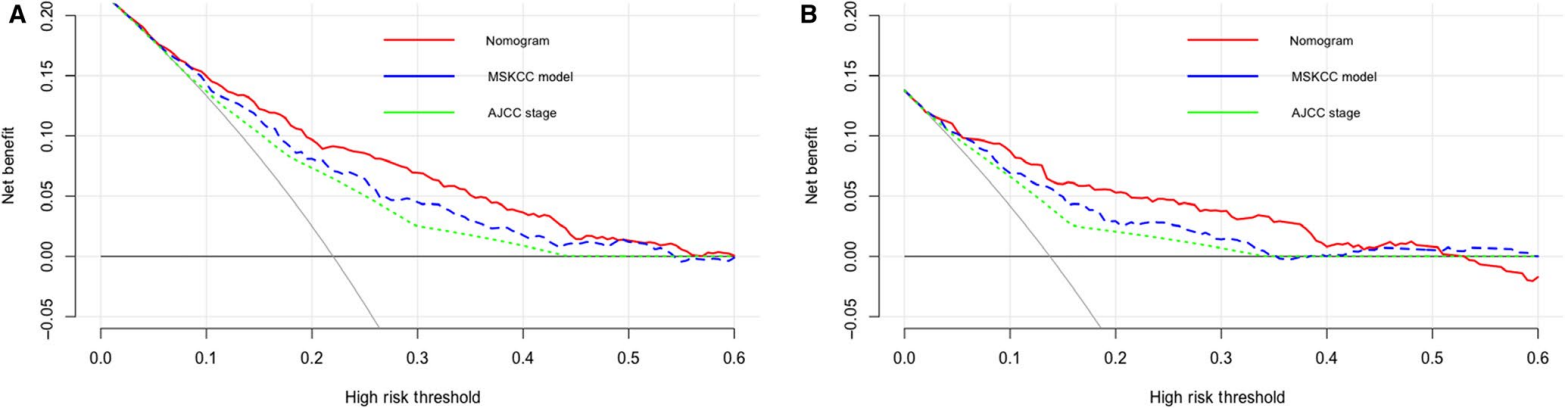
Decision curve analysis to assess the clinical usefulness of the nomogram, MSKCC model and AJCC staging in the primary cohort (A) and validation cohort (B). Dark gray line: assume no patients will die. Light gray line: assume all patients will die

Decision curve plots showed the nomogram was associated with improved clinical net benefits over the MSKCC model and AJCC stages (higher lines of prediction by the nomogram) within a practical range of threshold probabilities in both the primary cohort (Figure 3A) and the validation cohort (Figure 3B). Thus, the nomogram has the best clinical utilities in assessing individual prognosis among the three models.

### 3.6 Risk stratification based on nomogram scores

**FIGURE 4:**
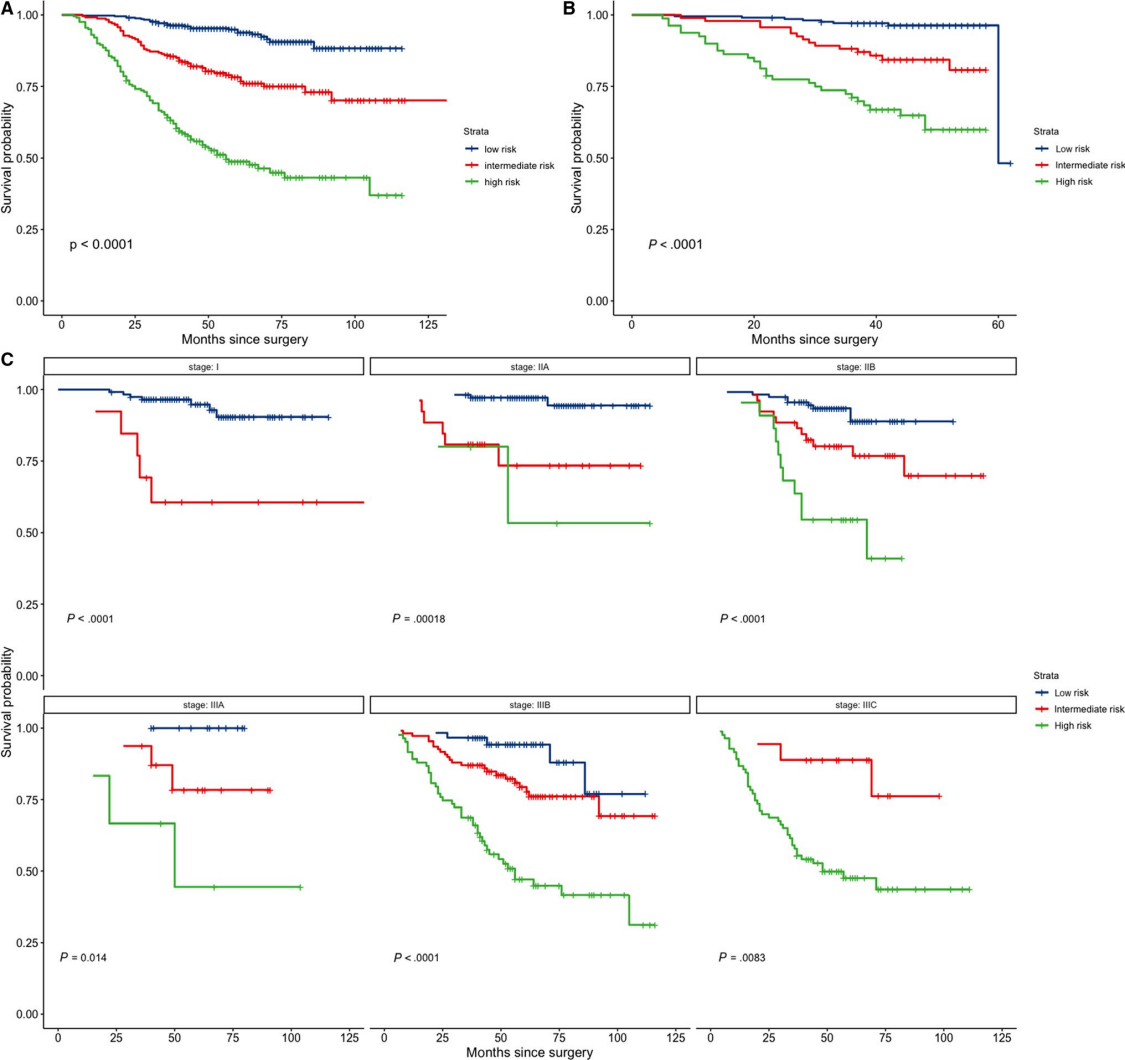
Kaplan‐Meier survival curves of risk groups stratified based on the nomogram for the primary cohort (A), the validation cohort (B), and within AJCC stages for the primary cohort. There are two few patients in the IIC stage to perform subgroup analysis

Patients were stratified into three risk groups based on nomogram derived risk scores with a recursive partitioning analysis. The subgroups were as follows: low‐risk group (risk score ≤12.68), intermediate‐risk group (12.68 < risk score ≤ 14.07), and high‐risk group (risk score >14.07). Kaplan‐Meier survival curve analysis showed the three groups had statistically different prognosis in both primary cohort (Figure 4A) and validation cohort (Figure 4B). Remarkably, this risk stratification could successfully discriminate patients with different prognosis within each AJCC stage (Figure 4C). There are only two patients in the IIC stage and subgroup analysis could not be performed. Besides, only two patients were categorized as high risk in the I stage and three patients were low risk in the IIIC stage. These patients were excluded from subgroup analysis because of the extremely small size.

## 4 DISCUSSIONS

In the present study, a nomogram was developed to estimate 3‐year and 5‐year survival probability for curatively resected nonmetastatic CRC patients. This nomogram outperformed the MSKCC model and AJCC TNM staging in terms of discrimination, calibration abilities, and clinical utilities. The nomogram was validated in a prospective cohort and demonstrated to be quite reliable.

The selection of factors in this study was based on their availability in routine practice and established associations with overall survival in previous publications.^12^, ^17^, ^18^ Not surprisingly, the nomogram incorporates the widely acknowledged independent prognostic factors such as T staging, N staging, histological grade, and pretreatment CEA levels. The endpoint for the nomogram is overall survival, which takes into account all cause of mortality. Age is well associated with all‐cause mortality, and thus also contributes to the nomogram. Notably, number of lymph nodes sampled is a major contributor to the nomogram. As early as 2003, a secondary analysis of the Intergroup Trial INT‐0089 trial demonstrated that an increase in number of lymph nodes examined was associated with increased survival for patients, regardless of nodal status.^19^ The more lymph nodes examined, the less likelihood of false negativity in nodal staging.^17^ The number of examined lymph nodes could relate to the surgical and pathological quality of treatment. Wide‐type KRAS/BRAF and microsatellite instability (MSI) have been associated with both increased lymph node yields and improved prognosis.^20^, ^21^ It is known that tumor microenvironment and the host's immune response are important in tumor progression, and a higher lymph node yield could reflect a stronger antitumor immune response. All in all, the number of lymph nodes retrieved might reflect the underlying biology. The more TLN, the more favorable biology it is. Another important contributor to the nomogram is GPS. GPS is calculated based on serum CRP and albumin levels. Increased serum CPR levels indicate systematic inflammation status and low serum albumin levels indicate malnutrition and cachexia. Both were associated with poor prognosis in various cancers. The GPS enables better appreciation of systematic inflammation and malnutrition and reflects tumor‐host interaction. Recent meta‐analysis of 41 studies with 9839 CRC patients showed GPS was a strong independent poor prognostic factor regardless of tumor stages.^12^

To date, there are four published nomograms predicting survival after radical surgery for nonmetastatic CRC. Two were developed from SEER database.^10^ Our nomogram outperformed one of the two modes—the MSKCC model.^10^ The other model was interested in making a reclassification of TNM staging and only investigated T staging and N staging.^3^ The third model was based on individual patient data from three phase 3 trials in Japan.^22^ Pretreatment CEA, TLN, and GPS were not investigated. The fourth model was the only model incorporating patients' systemic inflammatory status, but chose markers that were not as established as GPS or mGPS.^23^

The strengths of our nomogram include an appreciable size of representative patients in real clinical setting, a prospective validation cohort and readily available factors in routine practice. The variables used in the nomogram could be easily obtained by physicians in many community hospitals without any technical or cost barriers. Risk group stratification defined by the nomogram was a good complement to the 8th AJCC stage. The nomogram gives accurate and individualized mortality risk predictions and can discriminate different prognosis groups within the same TNM stage. It should enable improved patient counseling regarding treatment selection and follow‐up strategy. The nomogram itself is not intended to make treatment decisions, but the nomogram directed treatment strategy could be investigated in clinical trials.

There are several points should be addressed. First and foremost, this nomogram was developed from a cohort of patients treated at a single institution including only Chinese patients. Although internal validation and prospective external validation were performed to prevent over‐fit of current data, it would be better to validate the nomogram in patients from other institutions with diversified ethnicities. Second, important molecular factors such as KRAS/NRAS/BRAF and MSI were not investigated. These factors were good treatment efficacy predictors, but their prognostic roles were controversial. In a recent meta‐analysis, they were found to be not significantly or differentially associated with survival.^24^ Third, pretreatment CEA level was dichotomized after failing restrict cubic spline modeling. Although the cutoff value was verified to be reproducible in the validation cohort, the best way to categorize CEA is still not conclusive. Nonstandardized determination of CEA levels worldwide makes that more complex. Fourth, because the number of lymph nodes sampled relates to the quality of service CRC patients received and low lymph nodes yield might lead to understaging, we excluded patients with less than 12 sampled lymph nodes in the nomogram development to ensure reliability. Thus, the nomogram may not be transferrable to patients with less than 12 lymph nodes sampled. Fifth, the nomogram incorporated seven factors and that could possibly lead to model overfitting as a result of too many parameters. The gold way to avoid overfitting of a model is to retest it in a new set of data. The nomogram performed quite well in the independent validation cohort in terms of predictive discrimination, accuracy, and calibration ability, which means overfitting was not an issue. The selection of variables in the final model was based on the minimized AIC value. Every factor had incremental predictive ability. They all have established prognostic significance as shown by other publications.

## 5 CONCLUSIONS

In conclusion, we propose a nomogram that could provide individualized outcome predictions with good accuracy, reliability, availability, and applicability. It could be helpful to physicians and patients in the treatment decision‐making process.

## CONFLICT OF INTEREST

All authors declare no conflict of interest.

## AUTHOR CONTRIBUTIONS

Tingting hong and Xiaohong Wu designed the study. Tingting hong analyzed and interpretated the data and wrote the manuscript. Dongyan cai and Ying zhang collected the data. Dong hua, Linfang Jin, and Tingxun Lu provided valuable insights into data interpretation and manuscript writing.

## Data Availability

The datasets and the R codes used in the current study are available from the corresponding author upon reasonable request.
